# Genetic Dissection of Seed Storability and Validation of Candidate Gene Associated with Antioxidant Capability in Rice (*Oryza sativa* L.)

**DOI:** 10.3390/ijms20184442

**Published:** 2019-09-09

**Authors:** Zhiyang Yuan, Kai Fan, Laifu Xia, Xiali Ding, Li Tian, Wenqiang Sun, Hanzi He, Sibin Yu

**Affiliations:** National Key Laboratory of Crop Genetic Improvement, College of Plant Science and Technology, Huazhong Agricultural University, Wuhan 430000, Hubei, China

**Keywords:** seed viability, quantitative trait locus, fatty acid hydroxylase, oxidative stress, genotyping by sequencing, rice

## Abstract

Seed storability, defined as the ability to remain alive during storage, is an important agronomic and physiological characteristic, but the underlying genetic mechanism remains largely unclear. Here, we report quantitative trait loci (QTLs) analyses for seed storability using a high-density single nucleotide polymorphism linkage map in the backcross recombinant inbred lines that was derived from a cross of a *japonica* cultivar, Nipponbare, and an *indica* cultivar, 9311. Seven putative QTLs were identified for seed storability under natural storage, each explaining 3.6–9.0% of the phenotypic variation in this population. Among these QTLs, *qSS1* with the 9311 alleles promoting seed storability was further validated in near-isogenic line and its derived-F_2_ population. The other locus (*qSS3.1*) for seed storability colocalized with a locus for germination ability under hydrogen peroxide, which is recognized as an oxidant molecule that causes lipid damage. Transgenic experiments validated that a candidate gene (*OsFAH2*) resides the *qSS3.1* region controlling seed storability and antioxidant capability. Overexpression of *OsFAH2* that encodes a fatty acid hydroxylase reduced lipid preoxidation and increased seed storability. These findings provide new insights into the genetic and physiological bases of seed storability and will be useful for the improvement of seed storability in rice.

## 1. Introduction

Rice (*Oryza sativa* L.) is the staple food for more than half of the world’s population. Seed deterioration after harvest is a serious problem for rice production in Asia, especially for hybrid rice in the Southern area [1]. Rice seeds deteriorate during storage, resulting in reduction in germination rates, loss of seed viability, and a decline in nutritional quality. The loss of seed viability due to seed deterioration has been a great challenge to the crop production industry [2]. For example, an average annual 15 billion kilograms of rice grain yields in China is lost due to seed deterioration during storage [3]. Therefore, the improvement of seed storability to maintain seed viability during storage is becoming an important objective of rice and other crop breeding programs.

Seed storability is defined as the ability to remain alive during storage and is a vital trait for agricultural production and germplasm preservation in crops. Seed storability is a complex character affected by genetic and environmental factors during plant growth, seed maturation and post-harvest. It varies greatly among rice accessions that originate from different eco-geographic regions. For example, *indica*-type seeds maintained their viability longer than *japonica*-type seeds [4,5]. Using quantitative trait loci (QTLs) analysis and association mapping approaches, many research groups have identified numerous QTLs for seed storability in rice that were evaluated by natural storage or artificial aging [6,7,8,9,10,11,12,13]. Although there are several loci commonly detected across some populations, such as *qRGR-1* on chromosome 1, *qRGR3* on chromosome 3, *qLG-7* on chromosome 7, *qLG-9* on chromosome 9, and *qSS11* on chromosome 11, most QTLs identified for seed storability are different in various genetic populations. Thus far, *qLG-9*/*qSS-9* is the only locus that has been finely mapped [14,15]. Therefore, the genetic mechanisms of seed storability in rice remain largely unclear.

Multiple studies have shown that many physiological, biochemical, and metabolic alterations occur during seed storage. Reactive oxygen species (ROS) are often considered as a main source of seed deterioration caused by a decrease in seed viability [16]. Excessive ROS accumulation induced by storage environment could trigger oxidation and damage of membrane lipids, proteins, DNA and other molecules [17]. A long-lived ROS, hydrogen peroxide (H_2_O_2_), is recognized as an important signaling molecule and has strong oxidizing capacities, resulting in an oxidative stress that is associated with cellular component damage [18]. Lipid peroxidation is one of the most widely documented toxic effects of H_2_O_2_ on cellular components and biological molecules, especially polyunsaturated fatty acids (PUFA) in cell membranes. Some end products of PUFA peroxidation, such as malondialdehyde (MDA), could cause severe damage to cells by reacting with macromolecules when present at high levels, and are negatively correlated with seed vigor [19,20]. To control ROS-induced lipid peroxidation damage, seeds have detoxification mechanisms to scavenge or inactivate ROS. For example, lipoxygenases (LOX) are the important enzymes that catalyzed PUFA peroxidation in seed deterioration [21]. Seeds lacking *LOX3* or suppressing the gene, exhibited a low level of PUFA peroxidation [3,22,23], which led to enhanced seed storability in crops during storage. In addition, free fatty acids accumulation by lipid degradation during storage is one of the physiological causes of storage unstable. Fatty acid hydroxylase (FAH) acts as a major factor to α-hydroxylate free fatty acids and reduce the free fatty acid content [24]. However, whether lipid degradation mediated by FAH is associated with seed storability at the genetic level needs to be determined.

In this present study, a high-density single nucleotide polymorphism (SNP) linkage map was constructed in the backcross recombinant inbred line (BRIL) population, which was derived from a backcross of rice varieties Nipponbare (NIP) and 9311 to determine the genetic architecture of seed storability in rice and to detect QTLs associated with seed storability. Two loci for seed storability were finely mapped and confirmed using near-isogenic lines and its derived population, and one of them (*qSS3.1*) was colocalized with a locus for germination under hydrogen peroxide in the same population. Further transgenic tests identified a fatty acid hydroxylase gene as potential functional candidate conferring seed storability. These findings provide new insights in the physiological and genetic basis of seed storability in rice.

## 2. Results

### 2.1. Construction of High-Density Bin Map in BRILs

The BRIL population comprising 334 lines was genotyped using a genotyping-by-sequencing (GBS) approach and generated a total of 190.27 Gb of raw data, which was approximately 569.72 Mb for each line (Table S1). In total, 49,890 SNPs were detected in the population based on the Rice Genome Annotation Project (Release 7) and distributed evenly throughout the whole genome. A total of 2864 bins with the average size of 129.7 kb ranging from 1.7 kb to 7.3 Mb, were generated in the BRILs based on recombination breakpoints that occurred in each individual. A bin linkage map was constructed for the BRIL population (Table S1), spanning a total length of 1471.2 cM with an average interval of 0.53 cM between adjacent bins.

### 2.2. Seed Storability of the Parents and BRILs

**Figure 1:**
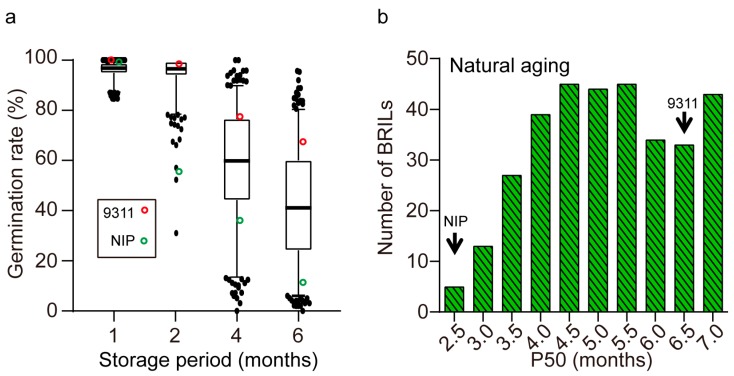
Seed germination of the parental lines and backcross recombinant inbred line (BRIL) population under natural storage. (**a**) Boxplot of germination rates after 1-, 2-, 4-, 6-month storage. The box edges indicate the range of the 25th to 75th percentiles with the mean value shown as the bold middle line. The whiskers represent the range of 5% to 95% of the data and outer dots are outliers. (**b**) Frequency distribution of seed storability as measured by P50, which is the time required for a reduction of viability to 50% under natural storage. Arrows indicate the means of parental lines Nipponbare (NIP) and 9311.

Seed germination (viability) was measured for the two parental lines and BRILs at several time-points, including one month (G1) after harvest and two months (G2), four months (G4), and six months (G6) during nature storage. Phenotypic variation of the parents and BRILs is illustrated in Figure 1. The mean of germination rate of almost all lines at G1 was greater than 95%. These results indicate that seeds of each line had high original seed viability and uniform initial quality (Figure 1a), which was suitable for evaluating seed storability. The BRIL population exhibited progressively decreased seed viability during six-month storage (Figure 1a). The average seed germination was 95% at G2 and fallen to 58.5% at G4 and 42.0% at G6. After four months of storage, the germination rate revealed large variation among lines. In terms of P50, which was measured as storage time in which viability declined to 50% of original viability, a transgressive performance beyond the parental values was observed in the population (Figure 1b). These results suggest large genetic variation for seed storability among the BRILs. In addition, two parental lines showed significant differences in the germination behaviors during storage. Germination rate of NIP seeds was reduced to 10%, while 9311 seeds remained 69% at G6 (Figure 1a). The parent 9311 seeds had a higher P50 value than NIP under natural storage (Figure 1b). These results indicate that 9311 seeds are more storable than NIP seeds.

### 2.3. QTLs for Seed Storability

**Figure 2:**
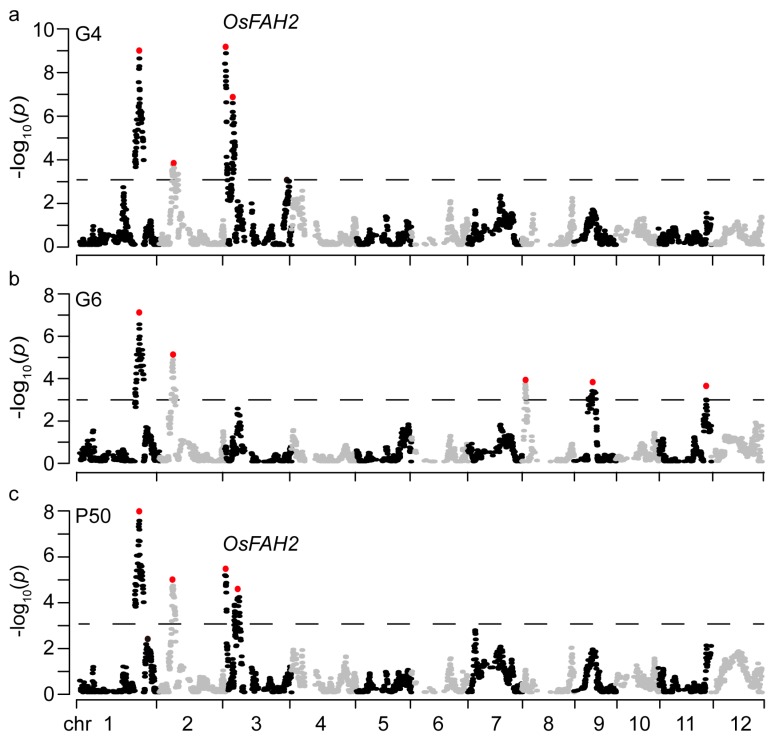
Manhattan plots of the loci for several parameters of seed storability. G4 at 4-m storage (**a**); G6 at 6-m storage (**b**); and P50 (**c**). The x-axis represents single nucleotide polymorphism (SNP) along each numbered chromosome; the y-axis represents the negative logarithm of the *p* value (-log_10_ *p*) for the SNP association. Horizontal dashed lines in the plots indicate the declaration thresholds. The main quantitative trait loci (QTLs) are highlighted by red dots.

To identify loci associated with seed storability, QTL analyses of seed viability at three time-points of G2, G4 and G6, and seed storability index P50 were conducted. A number of QTLs were identified for seed viability under natural storage (Table 1; Figure 2), except no QTL was detected for G2. Comparison of these QTLs among the assayed germination behaviors revealed that two QTLs (*qG4S1*, *qG4S2*) for G4 were repeatedly identified for G6, one QTL (*qSS3.1*) for P50 was found on the same location for G4 (Figure 2). Therefore, a total of seven QTLs distributed on chromosomes 1, 2, 3, 8, 9 and 11 were identified for seed storability parameters, each explaining 3.6% to 9.0% of the phenotypic variance in the population (Table 1). Of them, one (*qSS1*) was the commonly detected QTL across G4, G6, and P50, with the 9311 alleles enhanced seed storability. This is in consistent with correlation analysis, which revealed that seed storability (P50) was highly significantly correlated with the germination rates at G4 and G6 (*r*^2^ = 0.92, *p* < 0.01) among them in the BRIL population (Figure S1).

### 2.4. Validation of qSS1 for Seed Storability

**Figure 3:**
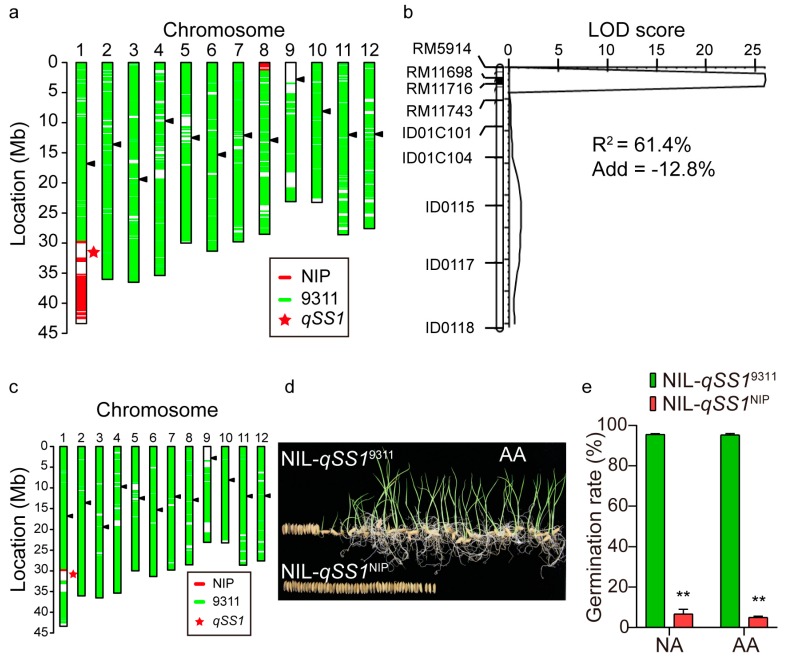
Validation of *qSS1*. (**a**) Graphic genotype of one line that carries an introduced NIP segment encompassing *qSS1*. Arrowhead (black) indicates centromeric region. (**b**) *qSS1* is narrowed down to the interval flanked by the markers RM11698 and RM11716 using a derived F_2_ population (*n* = 128). (**c**) Graphic genotype of NIL-*qSS1*^9311^. (**d, e**) Seed germination of NIL-*qSS1*^9311^ and NIL-*qSS1*^NIP^ after natural aging and artificial aging. NA and AA denote natural aging and artificial aging, respectively. Double asterisks in (**e**) indicate significant difference between NILs at *p* < 0.01.

To validate the *qSS1* effect, one line that carried an introduced NIP segment surrounding *qSS1* on chromosome 1 and another introduced segment on chromosome 8 within the 9311 background (Figure 3a) was crossed with 9311 to produce an F_2_ population. QTL analysis in the F_2_ population confirmed that *qSS1* located in the interval between RM11698 and RM11716 on chromosome 1 and had a major effect on seed storability, explaining 61.4% of the phenotypic variance (Figure 3b). A pairwise set of near-isogenic lines (NIL-*qSS1*^NIP^ and NIL-*qSS1*^9311^) carrying contrasting alleles at the *qSS1* region (less than 2 Mb) within the same 9311 background (Figure 3c) was developed by marker-aided selection from the F_2_ population. The germination rate of NIL-*qSS1*^NIP^ and NIL-*qSS1*^9311^ after artificial aging were 5.1% and 96.0%, respectively (Figure 3d,e). Consistently, seed viability of NIL-*qSS1*^NIP^ and NIL-*qSS1*^9311^ after natural aging for 12 months were 6.0% and 95.9%, respectively (Figure 3e). 

To fine map *qSS1* to a small region, five BRILs that carry the specific bin regions covering *qSS1* were further analyzed (Figure S2a). Of which, three lines carried the NIP alleles at bin region (B01C270) exhibited lower seed viability and seed storability than 9311 (*p* < 0.01). In contrast, the other two lines harbored the 9311 alleles at the QTL region revealed high seed storability as that of 9311 (Figure S2b). These results indicate that *qSS1* located on the bin B01C270 with approximately 23.9 kb (Figure S2c). In this interval, there are three annotated genes (Table S2). *Os01g55750* belongs to the Teosinte branched1/Cycloidea/Proliferating cell factor (TCP) family, *Os01g55740* encodes a rhomboid family protein, and *Os01g55730* encodes a function-unknown protein. 

### 2.5. QTLs for Germination Tolerance to Hydrogen Peroxide

Germination tests were also conducted for the G1 seeds under hydrogen peroxide. This treatment might mimic an excessive accumulation of hydrogen peroxide in seed deterioration during storage. The BRIL population revealed large variation in germination behavior under hydrogen peroxide (Figure S3a), suggesting the BRILs exhibited different antioxidant capability (AOC) or tolerance to oxidative stress. Germination rate under hydrogen peroxide was also significantly correlated with P50, although the coefficient was relatively low (Figure S1). Such correlations suggest that the genetic basis of seed storability might be associated with antioxidant capability. QTL analyses detected five loci associated with AOC (Figure S3b), which explained 30.6% of the phenotypic variance. Of them, a QTL (*qAOC3.1*) for antioxidant capability colocalized with *qSS3.1* for P50 on the peak bin B03C003 on chromosome 3 (Table 1), which is consistent with the positive correlation between these two parameters. 

### 2.6. Validation of qSS3.1

**Figure 4:**
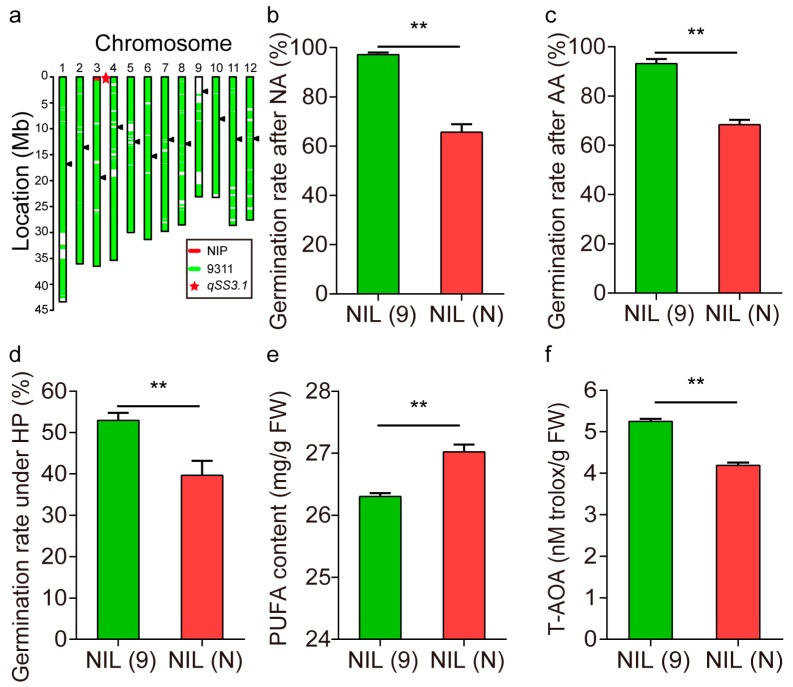
Comparison of the NILs that carry the contrasting alleles at *qSS3.1*. NIL (9) harbors the 9311 alleles, while NIL (N) carries the NIP alleles. (**a**) Graphic genotype of NIL (N) showing the introduction of a small fragment (red bar) that contains *qSS3.1* on the top of chromosome 3. Germination rate of the NILs after 6-m natural storage (NA) (**b**), artificial aging (AA) (**c**), and hydrogen peroxide (HP) (**d**). Polyunsaturated fatty acid (PUFA) content in the NILs (**e**), and total antioxidant activity (T-AOA) (**f**). Error bar represents mean ± SE with three replicates. Double asterisks denote significant difference at *p* < 0.01.

To confirm the effect of *qSS3.1* or *qAOC3.1*, a pairwise set of near-isogenic lines (NIL) was generated in the 9311 background carrying either NIP alleles (*qSS3.1*^NIP^) or 9311 alleles (*qSS3.1*^9311^) at the introduced *qSS3.1* segment (less than 700 kb) (Figure 4a), designated as NIL (N) and NIL (9), respectively. Comparison of the two NIL phenotype revealed that NIL (9) had a higher germination rate than NIL (N) under natural storage (Figure 4b). Notably, NIL (N) treated with artificial aging and hydrogen peroxide also displayed a significant reduction in germination compared with NIL (9) (Figure 4c,d). These results indicate that NIL (9) had better seed storage compared with NIL(N). Consistently, total antioxidant activity (T-AOA) was significantly higher in NIL (9), while PUFA content on average was significantly lower compared with NIL (N) (Figure 4e,f). These results suggest that the 9311 alleles at *qSS3.1* significantly promoted seed storability and were involved in antioxidant capability.

### 2.7. Candidate Gene for qSS3.1

Comparison of five BRILs that harbor the contrasting alleles surrounding *qSS3.1* revealed that *qSS3.1* located on the peak bin B03C003 (Figure S3c). This bin region is approximately 26.2 kb, containing eight predicted genes (Table S2). Among them, *Os03g01820* encodes a fatty acid hydroxylase (*OsFAH2*), the only gene controlling lipid degradation in this region. It was reported that *OsFAH2* catalyzed PUFA hydroxylation and reduced lipid peroxidation products during seed storage [25]. Thus, *OsFAH2* was a promising candidate for further analyses. Expression analysis revealed that *OsFAH2* was highly expressed at the reproductive stage and increased relative expression levels in NIL (9) compared with NIL (N) (Figure S4a,b). Sequence comparison revealed a one-base substitution causing an amino acid change (Ser in 9311 to Ala in NIP) in the first exon of the gene (Figure S4c). This finding strongly supports the candidacy of *OsFAH2* for seed storability.

**Figure 5:**
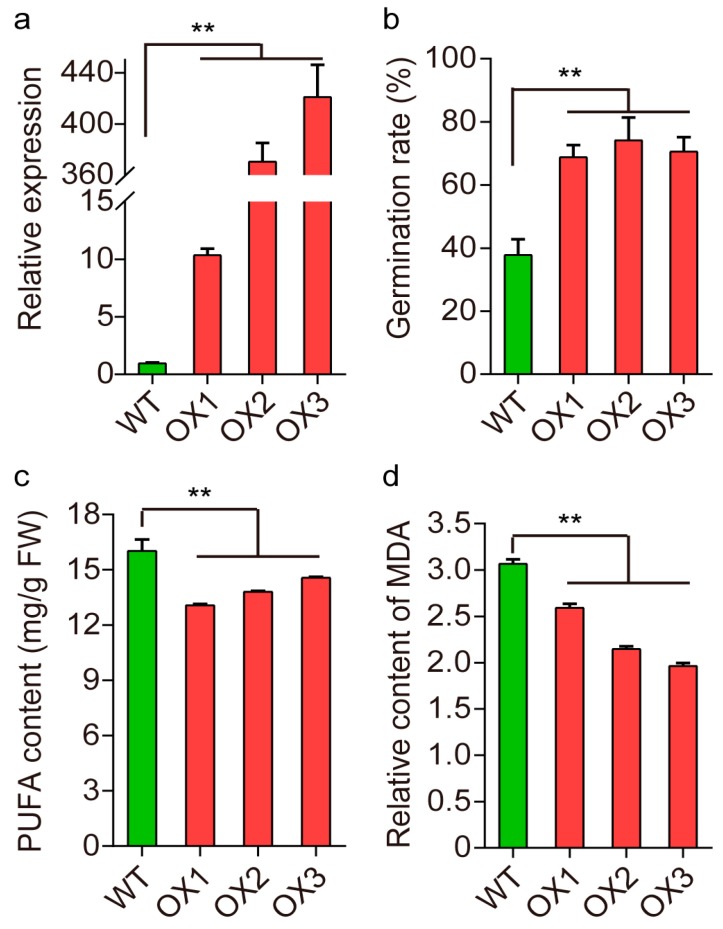
Germination behaviors of the transgenic lines overexpressing *OsFAH2* (**a**) Relative expression levels. (**b**) Germination rates after artificial aging. (**c**) Polyunsaturated fatty acid (PUFA) contents. (**d**) malondialdehyde (MDA) contents among the overexpression lines (OX1, OX2, OX3) and wild type (WT). Error bar represents mean ± SE with three to six replicates. Double asterisks indicate significant difference compared with WT at *p* < 0.01.

To further examine the role of *OsFAH2* in seed storability, *OsFAH2* from 9311 was overexpressed in the variety ZH11, which has poor seed storability similar to NIP. Three independent transformants (OX1, OX2, and OX3) showed significantly increased seed storability under artificial aging compared with wild type (Figure 5a,b). The transgenic lines overexpressing *OsFAH2* significantly reduced PUFA content compared with the wild type (Figure 5c). The content of MDA, an end product of lipid peroxidation was significantly reduced in the *OsFAH2* overexpression lines after artificial aging (Figure 5d). These results indicate that *OsFAH2* is the most likely gene for *qSS3.1* in which increased seed storability likely occurs through preventing lipid peroxidation. 

## 3. Discussion

### 3.1. High-Density Bin Map Developed in BRILs for QTL Detection

Seed storability is a crucial factor for viability maintenance during storage ensuring proper seedling establishment and high yield in crops. Many studies have shown that seed storability is a complex trait with remarkable variation controlled by polygenes [6,7,9,11,12,13]. In the present study, a high-density linkage map has been developed in the relative large population of NIP/9311, which harbors 2864 bins with an average physical interval of 129.7 kb (Table S1). In the BRIL population with a high-density genetic map and germination parameters at several time-points during natural storage, a number of loci associated with seed storability were identified, which explained the phenotypic variation range of 3.6–9.0%. This finding supports the notion that seed storability is a quantitative trait controlled by multiple genes. Almost all QTLs in the present study are mapped in the same or overlapping locations of those loci for seed storability or seed longevity in previous reports. For example, *qSS1* was located the same region of *qGP1/RGR1* in a previous study, which was detected in artificial aging and natural aging [10,13]. *qSS3.1* was mapped in the same region of *qGP3* [10], while *qSS3.2* resided in the similar region associated with seed viability [9]. *qG6S9* was likely near *qSS-9*/*qLG-9* that was finely mapped [14,15]. Comparison of the QTLs at various physiological states at present study revealed that two QTLs (*qSS1*, *qSS2*) were repeatedly identified across G4, G6 and P50, while the QTL (*qSS3.1*) was consistently detected for G4 and P50 and for AOC. These results indicate our identified QTLs in the BRILs with high-density bin map are robust and informative.

It is notable that seven loci for seed storability are identified in a small region less than 50 kb in the present investigation. This allowed to nominate candidate genes at the peak bin using the available gene annotation database. In particular, *qSS1* was validated by using NILs and a derived segregating population and located to a peak bin with a 23.9-kb region, where three genes were predicted. Among them, *Os01g55740* encoding a putative rhomboid homologue plays a key role in flower development [26]. *Os01g55730* encodes a function unknown protein. *Os01g55750*, named *OsTCP5*, has been reported in the modulation of phytohormone signaling in plants [27]. In *Arabidopsis*, *TCP14*, a homolog of *OsTCP5*, interacted with the protein GIBBERELLIN-ACID INSENSITIVE (GAI), act downstream of gibberellin mediate seed germination [28]. *OsTCP5* was up-regulated by strigolactones and negatively regulated mesocotyl length [29]. Hence, *OsTCP5* may be a putative candidate gene for *qSS1*. Another peak bin region harboring *qSS3.1* occupies the physical position of 0.48–0.51 Mb on the top of chromosome 3. There were eight predicted genes in this small region. It was difficult to pinpoint which candidate gene among them is for the QTL of interest. It was assumed that the QTL colocalization might share the same underlying causal gene (s). Therefore, QTL analysis was conducted for germination behavior under hydrogen peroxide to find if any QTLs for seed storability co-localized with the QTL region(s) for AOC. The results revealed both *qSS3.1* and *qAOC3.1* located in the same peak bin (B03C003). Based on this information, a fatty acid hydroxylase gene *OsFAH2* was nominated as the most likely candidate underlying the QTL, because *OsFAH2* is the only gene with a predicted function in lipids degradation in this QTL interval (Table 1, Figure S3), although other genes in the QTL region should not be excluded from consideration. Taken together, the BRILs with a high-density bin map and relative large population is an effective strategy to uncover novel QTLs for seed storability and can facilitate QTL dissection at a fine scale.

In addition, as a permanent genetic population, the genotype-defined BRILs also provide an excellent system for characterizing a variety of important traits, such as seed germination behaviors as noted in our study and yield, grain quality, stress tolerance, and water and nutrient use efficiency in other studies [30,31,32]. In particular, 9311 has been used as an elite parent of super hybrid rice and grown on a large scale in China. Therefore, considerably more QTLs with a fine resolution that spans a small number of candidate genes for agriculturally important traits in the BRIL population can be exploited immediately through marker-assisted selection for the improvement of superior rice production in breeding programs.

### 3.2. Seed Storability and Antioxidant Ability

Many protective and repair mechanisms involved in the physiological and biochemical properties have been proposed for the maintenance of seed viability during storage [33]. One of the protective mechanisms is associated with oxidation of cellular macromolecules, such as lipid and protein. However, the molecular and genetic regulations of seed storability still need to be elucidated.

Seed storability refers to the length of time a seed remains viable during natural storage. In general, it is time-consuming to determine the differences in seed storability during conventional storage. Instead, artificial aging with elevated ambient temperature and relative humidity was utilized to rapidly assess seed storability [6,8,13], could mimic truly molecular and biochemical events occurring during natural seed aging [34]. In the present study, germination behaviors under natural storage and artificial aging were measured. Seed germination tests under hydrogen peroxide were also conducted for the measurement of AOC, since hydrogen peroxide is recognized as a toxic molecule that could be used for artificial aging to some extent. Through investigations of overlapping QTLs, *qSS3.1* for P50 was colocalized with *qAOC3.1* for antioxidant capability or germination under hydrogen peroxide (Table 1). These results suggest a genetic connection between seed storability and antioxidant ability. Regarding the colocalization of *qSS3.1* and *qAOC3.1*, the overlap occurs in a small region of 26.2 kb that contains a fatty acid hydroxylase gene *OsFAH2* as the most likely candidate for the QTL of interest. Several lines of evidence from NILs and transgenic experiments further support that *OsFAH2* positively regulates seed storability and may function as an antioxidant by alleviating lipid peroxidation that occurs during storage. Firstly, the importance of the *qSS3.1* region was confirmed using near-isogenic lines that possess the single introduced NIP segment (700 kb) at the QTL. NIL (9) harboring the allele *qSS3.1*^9311^, remained a significant high seed viability under natural storage and artificial aging compared with NIL (N) carrying the allele *qSS3.1*^NIP^ (Figure 4). Secondly, it was reported that *OsFAH2* catalyzed PUFA hydroxylation and reduced lipid peroxidation products during seed storage [25]. To address whether or not the gene influences lipid peroxidation, antioxidant activities along with the indicators of lipid oxidation; FUFA and MDA were measured in seeds of the NILs. As expected, the antioxidant ability was much higher in NIL (9) than in NIL (N). The physiological parameters associated with lipid peroxidation in the NILs reveal that the 9311 alleles at *OsFAH2* reduced PUFA and MDA content compared with NIP alleles (Figure 4). Thirdly, *OsFAH2* was also highly expressed at the reproductive stage and increased relative expression levels in NIL (9) compared with NIL (N) (Figure S4). Finally, transgenic experiments were conducted to validate if the candidate gene controls AOC and seed storability. Transgenic experiments indicate that overexpression of *OsFAH2* could promote seed germination under both artificial aging and oxidizing stress, and reduced PUFA and MDA content compared with the wild type (Figure 5). These results lead us to conclude that *OsFAH2* was the most likely gene of *qSS3.1* and/or *qAOC3.1.* This gene may promote the antioxidant capability to prevent peroxidation of the cellular membrane, thus resulting in the maintenance of seed viability during storage.

## 4. Materials and Methods

### 4.1. Plant Materials

A backcross recombinant inbred line (BRIL) population was developed by single-seed descent from a backcross (BC_1_F_1_) of *japonica* cultivar Nipponbare (NIP) as donor and *indica* cultivar 9311 as the recurrent parent. The BRIL population was grown at the experimental field of Huazhong Agricultural University (HAU) in 2016 at Wuhan (30.48N, 114.2E), China. A pairwise set of NILs that carry the contrasting NIP and 9311 alleles at the target region within the 9311 background were generated using a marker-assisted backcross scheme.

### 4.2. Seed Storability Evaluation

Seeds for each BRIL were harvested at 35 days after heading under optimal conditions and equilibrated in a storage chamber with a low relative humility (30% RH) for 4 weeks. Then, seeds were divided into two parts for different treatments. One part (termed G1) was immediately used for germination tests. The other part was stored under warehouse storage conditions with room temperature 23–25 °C and relative humidity (RH) 65–75%. During storage, seed samples were taken out every two months for germination assays until the germination rate of the majority of lines was reduced to 0%. Three replicates of 50 seeds for each sample were placed on water-moistened filter papers and kept in a germination chamber at 25 °C for seven days under darkness. Germinated seeds were determined daily by examining the radicle protrusion. Seed germination rate or viability at defined time-points (2 months, 4 months, and 6 months) during storage was calculated as G2, G4 and G6, respectively. Seed storability was also expressed as P50, which is the time required for a reduction in viability to half the initial value (G1) using a sigmodial equation in Germinator software [35]. To measure seed storability rapidly, artificial aging experiments were conducted using a modified method [8], in which seed samples were treated at high temperature (43 °C) and RH (88%) for eight days in a thermostatic moisture regulator (HWS-080, Shanghai, China). In addition, germination tests under hydrogen peroxide were conducted to assay antioxidant capability as described above with a modification in which water-moistened filter papers were replaced by papers moistened with 300 mM hydrogen peroxide. All germination experiments were repeated three times for each sample. Significant differences in seed viability among the assayed lines were determined at *p* < 0.05 level using one-way analysis of variance (ANOVA).

### 4.3. Determination of Total Antioxidant Activities

Total antioxidant activity (T-AOA) was determined following an ABTS (2,2’-azinobis-3-ethylbenzothiazoline-6-sulfonic acid) decolorization assay [36]. One-gram of seed powder was homogenized in 9 mL of 0.9% sodium chloride buffer on ice and centrifuged at 12000 rpm at 4 °C for 15 min. Supernatant was used to measure the activities using a total antioxidant activity kit (A015–2, NJB Institute, Nanjing, China) according to the manufacturer’s instructions. Absorbance was measured by a spectrophotometer (Tecan Spark™ 10M, Männedorf, Switzerland) at a wavelength of 405 nm. T-AOA values were expressed as ABTS radical scavenging activity relative to the activity of Trolox as a standard.

### 4.4. Measurement of Relative Electrolyte Leakage and MDA Content

Relative electrolyte leakage was determined according to the method with minor modifications [37]. Briefly, the de-husked seeds were immersed in double distilled water for 24 h. An initial conductivity (R1) was measured with a digital conductometer (FE30, Mettler Toledo, Switzerland). Then, the conductivity (R2) was determined after the seeds were boiled for half an hour. Relative electrolyte leakage was calculated as the ratio of R1 to R2.

MDA content was determined according to the reported method [38] with minor modifications. The seed powder was homogenized in 10% trichloroacetic acid, and centrifuged at 12,000 *g* for 10 min. An equal volume of 0.67% thiobarbituric acid was added to the supernatant, and the mixture was heated in boiling water for half an hour. After centrifuging, the absorbance of the supernatant was determined at 450, 532, and 600 nm. MDA content was calculated using the following formula:

MDA content (μmol/L) = 6.45 × (OD_532_ − OD_600_) − 0.56 × OD_450_. (1)

### 4.5. Total PUFA Measurement Using the GC-MS Method

Fatty acids in seeds were extracted, as described previously [39] and measured using a gas chromatography-mass spectrometer (GC-MS) (QP2010ULTRA, Shimadzu, Japan) with a chromatographic RESTEK Rtx Wax column. Seed samples (0.2 g) were immersed in 1.5 mL methanol containing 5% H_2_SO_4_, 100 μL glyceryltriheptadecanonate and 0.01% butylated hydroxytoluene in tubes. The tubes were incubated in a water bath at 85 °C for 3 h. After cooling to room temperature, hexane (2 mL) and double distilled water (2 mL) was added, mixed, and set at room temperature for 15 min. After centrifuging at 3000 *g* for 10 min, the upper phase was transferred to a new glass tube. The fatty acid methylester solutions were injected to GC through the injection port with detector temperature at 280 °C and an oven temperature at 180 °C for 2 min. Then the temperature increased by 10 °C/min up to 220 °C for 5 min. Fatty acid content was quantified based on the added internal standard heptanoic acid (17:0). Total PUFAs in seeds were the sum of C16:1, C18:1, C18:2, C18:3 and C20:1.

### 4.6. DNA Extraction and SNP Genotyping

Genomic DNA was extracted from young seedling leaves using the CTAB method [40]. The genotypes of the BRILs were analyzed using a genotyping-by-sequencing (GBS) strategy, as described previously [41] with minor modifications. Genomic DNA was digested with the restriction enzyme *Mse*I (Thermo Scientific, Madison, WI, USA), and unique barcode adapters were added to each sample well. Barcode adapters were designed and modified according to the standard Illumina adapter design for paired-end read libraries. The ligation reaction was incubated 1 h at 22 °C with T4 DNA ligase (Thermo Scientific, Madison, WI, USA) and inactivated at 65 °C for 20 min. Twenty-four ligation products for different samples were pooled in a single tube, and the products were amplified with 10 cycles of PCR. The amplified library was purified using a QIA quick PCR purification kit (Qiagen, Hilden, Germany), quantified on an Agilent 2100 Bioanalyzer (Agilent Technologies, Palo Alto, CA, USA), and finally sequenced on an Illumina HiSeq 2000 instrument (Illumina, San Diego, CA, USA).

The sequencing reads were aligned to the Nipponbare genome sequence (IRGSPv6) using SOAP2 software (version 2.20) [42]. SNP calling was performed with realSFS based on the Bayesian estimation of site frequency at every site. All SNPs were filtered using a Practical Extraction and Report Language (PERL) script based on the following criteria: loci with >50% missing data and minor allele frequency less than 5% in the population.

Based on SNP genotyping, bin was defined by a unique overlapping recombination segment across the BRILs according to a previously reported approach [43]. A bin without breakpoints was generated using R/qtl package function *fill.geno* with the ‘‘argmax’’ method. The high-density bin map was constructed as previously described [44]. Briefly, the sliding window approach was adopted to evaluate a group of consecutive SNPs for genotyping and determination of recombination breakpoints along chromosomes of each individual. Blocks with length less than 250 kb in which the number of sequenced SNPs was fewer than five were masked as missing data to avoid false double recombination. Genotypes of bins for regions at the transitions between two different genotype blocks were imputed using R/qtl package. The genetic linkage map was constructed using the R/qtl package function *est.map* with Haldane map method [45]. The genotype data of the BRIL population are available on request.

### 4.7. QTL Analyses

Germination rates at G2, G4 and G6 during storage relative to G1 were calculated for each BRIL. To improve the normality of the frequency distribution of germination rates in the population, germination percentage (x) at three storage points (G2, G4 and G6) and germination under hydrogen peroxide for each BRIL was transformed by arcsin (x)^0.5^. QTL analyses of the relative germination data and the transformed data with bins as markers were performed using composite interval mapping in QTL Cartographer V2.5 [46]. The thresholds of logarithm of odds (LOD) or *p* values were determined with 1000 permutations to declare the presence of QTL in a given bin. If several adjacent bins showed LOD values greater than the threshold, a QTL was tentatively located in the most significant bin (peak bin) with the highest LOD value.

### 4.8. Vector Construction and Rice Transformation

To construct the overexpression vector, *OsFAH2* was amplified from the cDNA of 9311 using specific primers (Table S3 and cloned into the linearized *p*CAMBIA1301S [47] with *Kpn*I using the Seamless Assembly and Cloning Kit (Aidlab Co., Beijing, China). The resultant construct was transferred into the *japonica* rice Zhonghua11 using the transformation method [48]. Transgenic plants were evaluated for seed storability and physiological changes as described above. All primers used for the transgenic experiments are listed in Table S3.

### 4.9. Quantitative Real-Time PCR Analysis

Total RNA from flag leaf at flowering stage was extracted using a TRIzol Reagent Kit (Invitrogen, Carlsbad, CA, USA). *OsFAH2* expression was detected using specific primers also listed in Table S3. Quantitative real-time PCR analysis was conducted using SYBR Green Master (Roche Diagnostics, Mannheim, Germany) with the ABI 7500 Real-Time PCR System. The rice *ubiquitin* gene was used as the internal control, and expression levels of the assayed genes were analyzed as described [49].

## 5. Conclusions

In conclusion, the BRIL population of NIP/9311 with high-density bin map was used to identify several QTLs and potential candidate genes for seed storability. Moreover, *OsFAH2* associated with AOC influence seed storability. These results provide an opportunity for in-depth investigations into the molecular mechanisms for seed viability loss during storage. These findings offer novel insights into understanding the physiological and genetic nature of seed storability and will be beneficial for marker-assisted breeding to improve seed storability properties.

## Figures and Tables

**Table 1:** Quantitative trait loci (QTLs) identified for seed storability and antioxidant capability in the 9311/ Nipponbare (NIP) backcross recombinant inbred lines (BRILs).

Trait	QTL	Chr	Bin	Start (Mb)	Bin Size (kb)	Add	LOD	PVE (%)	Candidate Gene
G4	*qG4S1*	1	B01C270	32.09	23.93	-0.09	8.7	9.0	
	*qG4S2*	2	B02C083	7.32	47.8	-0.06	3.6	3.6	
	*qG4S3.1*	3	B03C003	0.48	26.21	-0.09	8.7	9.0	*OsFAH2*
	*qG4S3.2*	3	B03C058	4.25	62.7	0.08	6.5	6.7	
G6	*qG6S1*	1	B01C270	32.09	23.93	-0.08	7.0	8.5	
	*qG6S2*	2	B02C083	7.32	47.8	-0.08	5.0	5.9	
	*qG6S8*	8	B08C021	1.73	114.23	0.07	3.7	4.3	
	*qG6S9*	9	B09C054	9.97	153.97	-0.07	3.7	4.4	*OsTPP7*
	*qG6S11*	11	B11C210	25.84	19.6	0.06	3.5	4.2	
P50	*qSS1*	1	B01C270	32.09	23.93	-0.39	7.9	8.7	
	*qSS2*	2	B02C078	7.03	78.64	-0.34	4.9	5.2	
	*qSS3.1*	3	B03C003	0.48	26.21	-0.33	5.3	5.7	*OsFAH2*
	*qSS3.2*	3	B03C094	6.84	22.69	0.37	4.5	4.8	
AOC	*qAOC1*	1	B01C345	38.37	41.05	-0.06	6.7	6.8	*SD1*
	*qAOC3.1*	3	B03C003	0.48	26.21	-0.08	8.9	9.0	*OsFAH2*
	*qAOC3.2*	3	B03C265	34.31	32.75	0.06	3.9	4.0	
	*qAOC4*	4	B04C113	20.11	49.21	-0.04	3.0	3.0	
	*qAOC11*	11	B11C106	10.91	36.49	-0.05	3.5	3.4	

Seed storability, measured by three indexes: germination rates after the 4-month (G4) and 6-month (G6) storage, and P50, the time required for a reduction of viability to 50% under natural storage; AOC: Antioxidant capability; Chr: Chromosome. Start: Physical position of a given bin based on the Rice Genome Annotation Project (Release 7). Bin size: size of the peak bin harboring a QTL. Add and PVE (%) indicate additive effect and phenotypic variance explained by a given QTL, respectively. The positive Add value represents the NIP alleles increased the effect.

## Supplementary Materials

Supplementary materials can be found at https://www.mdpi.com/1422-0067/20/18/4442/s1. Figure S1. Correlation among germination behaviors and antioxidant capability; Figure S2. Effect of the peak bin harboring qSS1; Figure S3. QTL analysis for germination rate in the BRILs under hydrogen peroxide and the effect of the peak bin harboring qSS3.1; Figure S4. Comparison of sequence variation and expression of OsFAH2 between NIP and 9311; Table S1. Distribution of Bins in chromosomes in the BRILs; Table S2. Candidate genes in the peak bin harboring the QTL of interest; Table S3. Primers used in this study.

## Abbreviations

BRIL	Backcross Recombinant Inbred Line
AA	Artificial Aging
AOC	Antioxidant Capability
FAH	Fatty Acid Hydroxylase
GBS	Genotyping by Sequencing
LOX	Lipoxygenases
LOD	Logarithm of Odds
MDA	Malondialdehyde
NA	Natural Aging
NIL	Near-isogenic Line
PUFA	Polyunsaturated Fatty Acids
PVE	Phenotypic Variance Explained
QTLs	Quantitative Trait Loci
ROS	Reactive Oxygen Species
SNP	Single Nucleotide Polymorphism
SS	Seed Storability
T-AOA	Total Antioxidant Activity
TCP	Teosinte branched1/Cycloidea/Proliferating cell factor
