# Supplementary material for: Genetic Dissection of Seed Storability and Validation of Candidate Gene Associated with Antioxidant Capability in Rice (Oryza sativa L.)
Source: Int J Mol Sci. 2019 Sep 9;20(18):4442. doi: 10.3390/ijms20184442 (PMC6770242; doi:10.3390/ijms20184442)
Supplement: Supplementary file 1 [file ijms-20-04442-s001.pdf]

Genetic Dissection of Seed Storability and Validation of Candidate Gene Associated with Antioxidant Capability in Rice (*Oryza sativa* L.)

Zhiyang Yuan [1], Kai Fan [1], Laifu Xia [1], Xiali Ding [1], Li Tian [1], Wenqiang Sun [1], Hanzi He [1] and Sibin Yu [1,*]

[1] National Key Laboratory of Crop Genetic Improvement, College of Plant Science and Technology, Huazhong Agricultural University, Wuhan 430000, Hubei, China; yuanzhiyang1989@163.com (Z.Y.); 1500595632@qq.com (K.F.); 2609754206@qq.com (L.X.); 728217547@qq.com (X.D.); litian_ccc@163.com (L.T.); wqsun@webmail.hzau.edu.cn (W.S.); hzhe@mail.hzau.edu.cn (H.H.)

* Correspondence: ysb@mail.hzau.edu.cn

Supplementary Figures and Tables

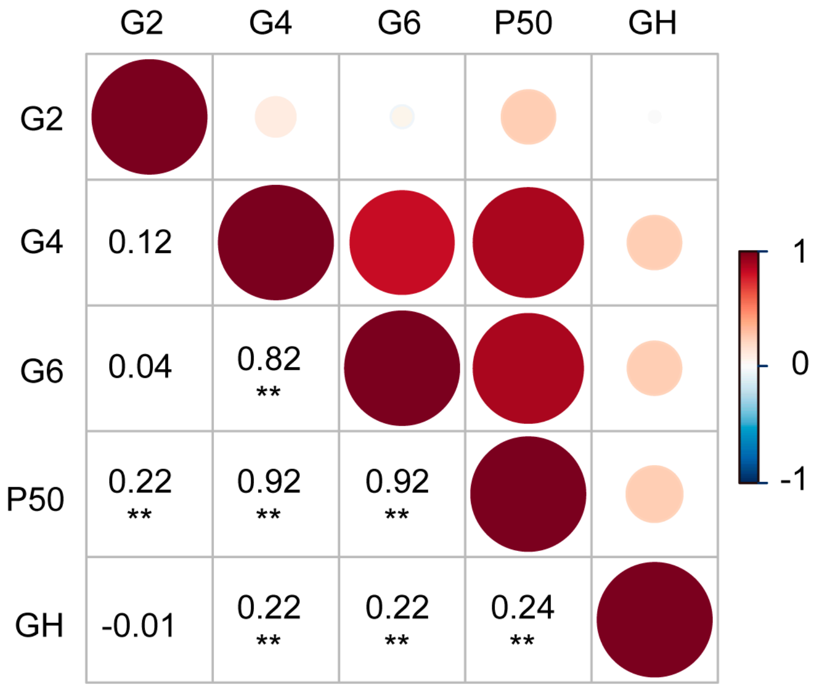

Figure S1. Correlation among germination behaviors and antioxidant capability. Heatmap of Pearson's correlation coefficients among seed storability parameters (means in triangle) in the BRILs. G1, G2, G4, and G6 represents germination rates after 1-, 2-, 4-, 6-month storage, respectively. P50 is the time required for a reduction of viability to 50% under natural storage. GH indicates germination rate under hydrogen peroxide. Asterisks indicate significant correlations using a two-tailed t-test ($p <$ 0.01).

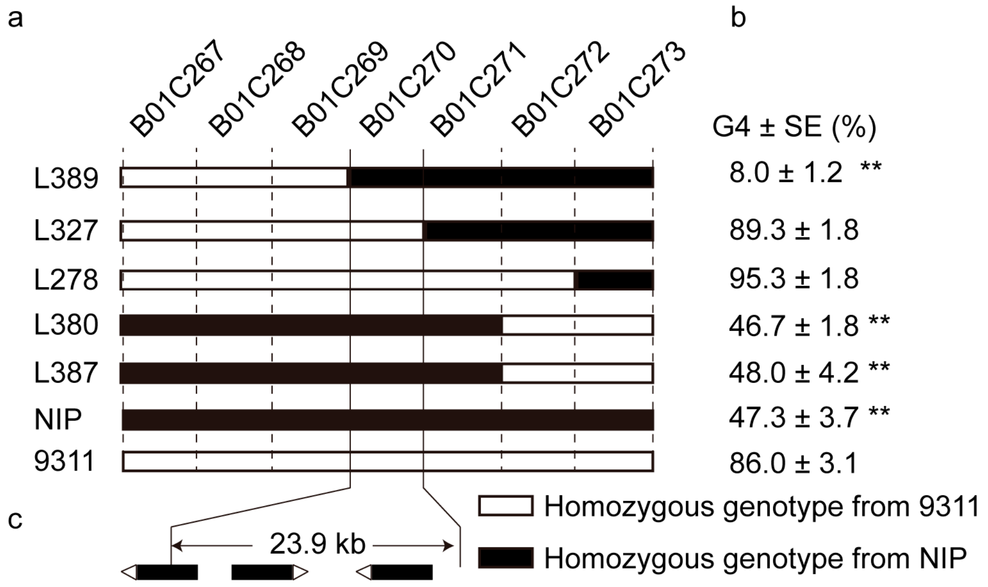

Figure S2. Effect of the peak bin harboring *qSS1*. (**a**) Genotypes of five selected BRILs and the parents (NIP and 9311). Black rectangle represents the homozygous NIP genotype, and white rectangle shows the homozygous 9311. (**b**) G4 ± SE, means of germination rate at G4 with three replicates. Double asterisks in the left panel indicate significant difference compared with 9311 at $p < 0.01$. (**c**) Three annotation genes in the peak bin for *qSS1*.

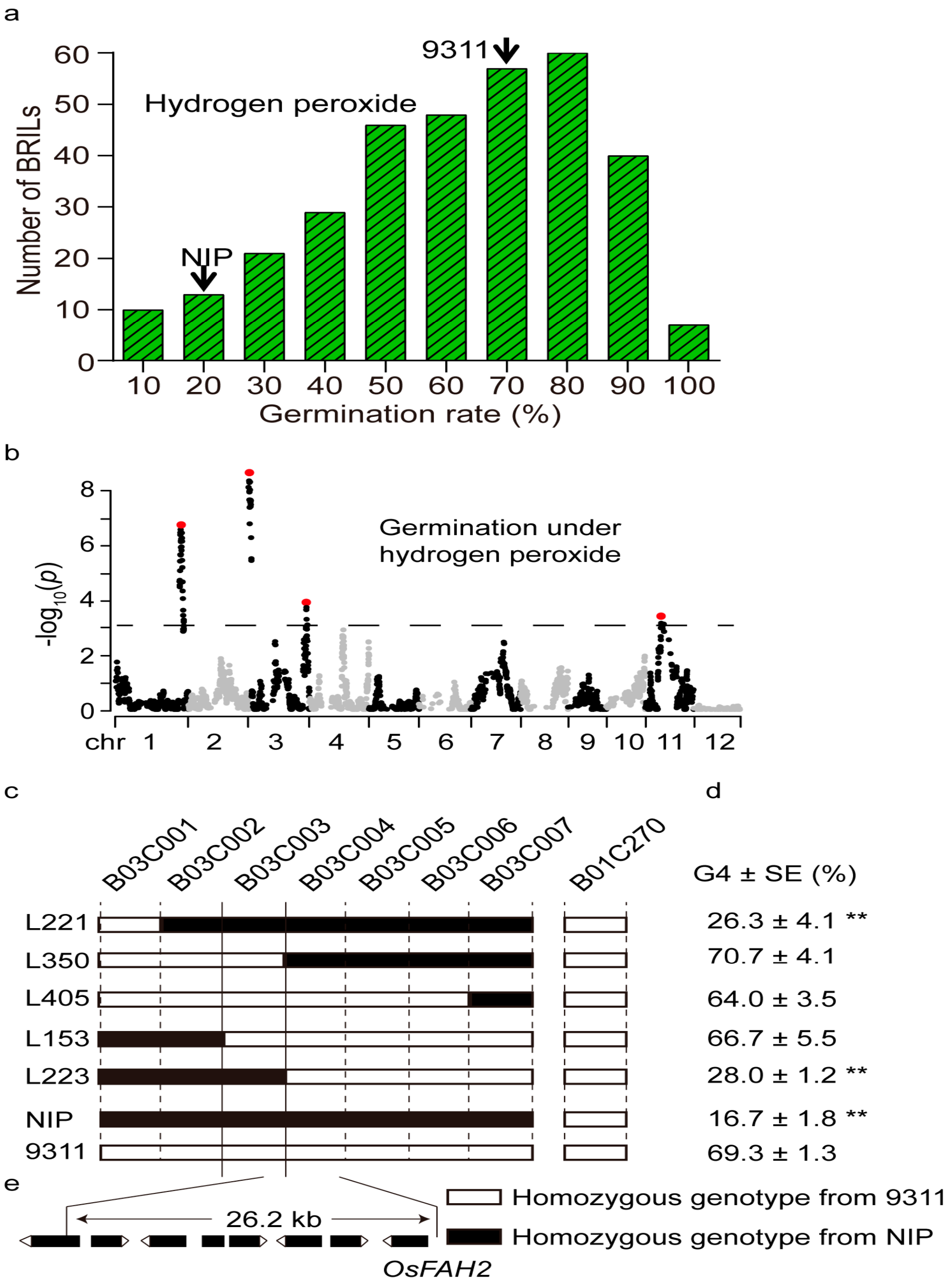

Figure S3. QTL analysis for germination rate in the BRILs under hydrogen peroxide and the effect of the peak bin harboring *qSS3.1*. (**a**) Frequency distribution of germination rate of the G1 seeds under hydrogen peroxide. Arrows indicate the means of parental lines NIP and 9311. (**b**) Manhattan plot of QTL analysis for the germination rate under hydrogen peroxide. (**c**) Graphical genotypes of five BRILs and the parents. Black rectangle represents the homozygous NIP, and white rectangle shows the homozygous 9311. (**d**) Means of seed germination rate of five BRILs and parents at G4 with three replicates. Double asterisks indicate significant difference compared with 9311 at $p < 0.01$. (**e**) Candidate genes for *qSS3.1* in the peak bin in Table 1.

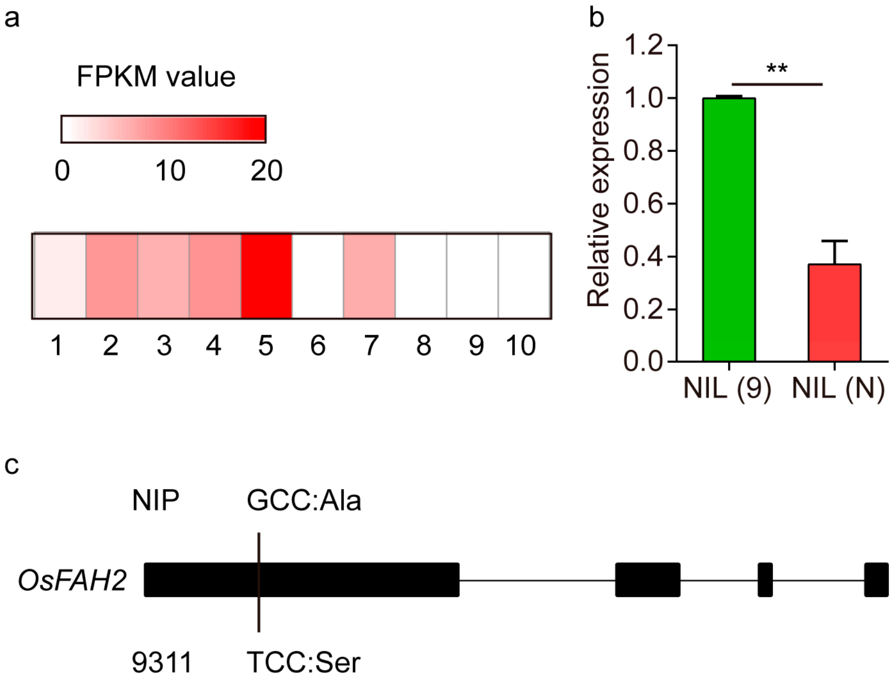

Figure S4. Comparison of sequence variation and expression of *OsFAH2* between NIP and 9311. (**a**) Expression level in different tissues of NIP. 1, Young leaf; 2, Shoot; 3, Pre-emergence inflorescence; 4, Postemergence inflorescence; 5, Pistil; 6, Anther; 7, Seed at 5 days after pollination (DAP); 8, Seed at 10 DAP; 9, Embryo at 25 DAP, 10, Endosperm at 25 DAP. (**b**) Relative expression of *OsFAH2* in NIL (9) and NIL(N). Error bar represents mean ± SE with three replicates. Double asterisks indicate significant difference at $p < 0.01$. (**c**) Schematic gene model showing a nonsynonymous mutation occurred in the coding region of *OsFAH2* in NIP and 9311.

Table S1. Distribution of Bins in chromosomes in the BRILs.

Chromosome	Bin Number	Average Distance between Adjacent Bin (cM)	Bin Size (kb)		
			Mean	Min	Max
1	423	0.48	102.04	3.79	2,556.59
2	363	0.46	98.16	1.71	2,498.17
3	289	0.61	125.43	6.53	3,881.13
4	284	0.49	124.88	5.50	5,862.09
5	277	0.47	106.91	4.31	3,070.50
6	125	0.72	249.68	11.95	5,098.52
7	210	0.54	140.45	3.89	1,973.62
8	154	0.60	183.89	2.59	7,340.89
9	182	0.50	125.27	4.22	2,123.23
10	149	0.45	155.68	7.33	4,079.71
11	228	0.53	126.71	5.22	2,902.89
12	180	0.51	152.69	6.41	2,204.44
Total	2,864	0.53	129.67	1.71	7,340.89

Table S2. Candidate genes in the peak bin harboring the QTL of interest.

QTL	Locus Name	Annotation Gene
qSS1	LOC_Os01g55730	AGAP003732-PA
	LOC_Os01g55740	OsRhmbd4 - Putative Rhomboid homologue
	LOC_Os01g55750	TCP family transcription factor
qSS3.1	LOC_Os03g01750	Dual specificity protein phosphatase
	LOC_Os03g01760	Exostosin family domain containing protein
	LOC_Os03g01770	Rhodanese
	LOC_Os03g01780	Transposon protein
	LOC_Os03g01790	Zinc finger, C3HC4 type domain containing protein
	LOC_Os03g01800	Glycosyl hydrolases family 16
	LOC_Os03g01810	SNF7 domain containing protein
	LOC_Os03g01820	Fatty acid hydroxylase

Table S3. Primers used in this study.

Primer	Sequence (5'-3')	Purpose
OsFAH2-pc1301s	Forward: atgatgatgataaaggtaccatgctcccgtacgcgacggcggcgg	OX vector
	Reverse: ctagaggatccccgggtaccatcttgtttcccgttgccgaatcca	
Hn	Forward: acggtgtcgtccatcacagtttgcc	Positive test
	Reverse: ttccggaagtgcttgacattgggga	
Ubq	Forward: aaccagctgaggcccaaga	qRT PCR
	Reverse: acgattgatttaaccagtccatga	
OsFAH2	Forward: catctacgggactgacagaggc	qRT PCR
	Reverse: cttgtttcccgttgccgaatcc	
ID0115	Forward: tgtttgttccagtggatctg	Mapping
	Reverse: accaaaacacgtttatgcaa	
ID0117	Forward: gtccatgaaattgtgaccaa	
	Reverse: acaaaatgggtccaaaagg	
ID0118	Forward: tagtgtcgaagcctttgctc	
	Reverse: ttcaaaatcagcgtcagttc	

ID01C101	Forward: ttttgtgcgaaaactttttat
	Reverse: gaccacatcgaatatgcttt
ID01C104	Forward: ggagagcaagagcagtagtt
	Reverse: acgaggtactacgacttgatg
RM5914	Forward: tgtgataagggatgcacttgagc
	Reverse: caagatctccacatttgactcacg
RM11698	Forward: atcagcatcccaaagctagaacc
	Reverse: aaccgtatattgagggagcaagc
RM11716	Forward: cctctacctcgcccaacagc
	Reverse: gaggaccgactccctgatcg
RM11743	Forward: aaggtcaaggaaacagggactgg
	Reverse: agccacgaattccactttcagc
